# ^68^Ga-Prostate-specific membrane antigen (PSMA) positron emission tomography (pet) in prostate cancer: a systematic review and meta-analysis

**DOI:** 10.1590/S1677-5538.IBJU.2019.0817

**Published:** 2020-11-10

**Authors:** Cristina S. Matushita, Ana M. Marques da Silva, Phelipi N. Schuck, Matteo Bardisserotto, Diego B. Piant, Jonatas L. Pereira, Juliano J. Cerci, George B. Coura, Fabio P. Esteves, Barbara J. Amorim, Gustavo V. Gomes, Ana Emília T. Brito, Wanderley M. Bernardo, Eduardo Mundstock, Stefano Fanti, Bruna Macedo, Diego H. Roman, Cinthia Scatolin Tem-Pass, Bruno Hochhegger

**Affiliations:** 1 Pontifícia Universidade Católica do Rio Grande do Sul Instituto do Cérebro do Rio Grande do Sul Porto AlegreRS Brasil Instituto do Cérebro do Rio Grande do Sul, Pontifícia Universidade Católica do Rio Grande do Sul - PUCRS, Porto Alegre, RS, Brasil; 2 Pontifícia Universidade Católica do Rio Grande do Sul Faculdade de Ciências Laboratório de Imagens Médicas Porto AlegreRS Brasil Laboratório de Imagens Médicas, Faculdade de Ciências, Pontifícia Universidade Católica do Rio Grande do Sul - PUCRS, Porto Alegre, RS, Brasil; 3 Clínica Kozma Balneário CamboriúSC Brasil Clínica Kozma - Balneário Camboriú, SC, Brasil; 4 Instituto Hospital Erasto Gaertner CuritibaPR Brasil Instituto Hospital Erasto Gaertner, Curitiba, PR, Brasil; 5 Quanta Diagnóstico e Terapia CuritibaPR Brasil Quanta Diagnóstico e Terapia, Curitiba, PR, Brasil; 6 Instituto do Câncer de São Paulo Departamento de Medicina Nuclear São PauloSP Brasil Departamento de Medicina Nuclear, Instituto do Câncer de São Paulo, São Paulo, SP, Brasil; 7 OncoPETscan BlumenauSC Brasil OncoPETscan, Blumenau, SC, Brasil; 8 Universidade Estadual de Campinas Departamento de Medicina Nuclear CampinasSP Brasil Departamento de Medicina Nuclear, Universidade Estadual de Campinas - UNICAMP, Campinas, SP, Brasil; 9 Núcleos Centro de Medicina Nuclear GuaráSP Brasil Núcleos Centro de Medicina Nuclear, Guará, SP, Brasil; 10 Real Hospital Português de Beneficência PernambucoPE Brasil Real Hospital Português de Beneficência, Pernambuco, PE, Brasil; 11 USP Faculdade de Medicina Programa de Pós-Graduação em Medicina São PauloSP Brasil Programa de Pós-Graduação em Medicina, Faculdade de Medicina - USP, São Paulo, SP, Brasil; 12 Pontifícia Universidade Católica do Rio Grande do Sul Faculdade de Medicina Programa de Pós-Graduação em Saúde da Criança Porto AlegreRS Brasil Programa de Pós-Graduação em Saúde da Criança, Faculdade de Medicina, Pontifícia Universidade Católica do Rio Grande do Sul - PUCRS, Porto Alegre, RS, Brasil; 13 University of Bologna Diagnostic and Specialized Medicine Department of Experimental Bologna Italy Department of Experimental, Diagnostic and Specialized Medicine-DIMES, University of Bologna, Bologna, Italy

**Keywords:** Prostate cancer, familial [Supplementary Concept], Glu-NH-CO-NH-Lys-(Ahx)-((68)Ga(HBED-CC)) [Supplementary Concept], (225)Ac-PSMA-617 [Supplementary Concept]

## Abstract

**Introduction:**

Prostate cancer (PC) is the second most commonly diagnosed cancer in males. ^68^Ga-PSMA PET/CT, a non-invasive diagnostic tool to evaluate PC with prostate-specific membrane antigen (PSMA) expression, has emerged as a more accurate alternative to assess disease staging. We aimed to identify predictors of positive ^68^Ga-PSMA PET and the accuracy of this technique.

**Materials and methods:**

Diagnostic accuracy cross-sectional study with prospective and retrospective approaches. We performed a comprehensive literature search on PubMed, Cochrane Library, and Embase database in search of studies including PC patients submitted to radical prostatectomy or radiotherapy with curative intent and presented biochemical recurrence following ASTRO 1996 criteria. A total of 35 studies involving 3910 patients submitted to 68-Ga-PSMA PET were included and independently assessed by two authors: 8 studies on diagnosis, four on staging, and 23 studies on restaging purposes. The significance level was **α**=0.05.

**Results:**

pooled sensitivity and specificity were 0.90 (0.86-0.93) and 0.90 (0.82-0.96), respectively, for diagnostic purposes; as for staging, pooled sensitivity and specificity were 0.93 (0.86-0.98) and 0.96 (0.92-0.99), respectively. In the restaging scenario, pooled sensitivity and specificity were 0.76 (0.74-0.78) and 0.45 (0.27-0.58), respectively, considering the identification of prostate cancer in each described situation. We also obtained specificity and sensitivity results for PSA subdivisions.

**Conclusion:**

^68^Ga-PSMA PET provides higher sensitivity and specificity than traditional imaging for prostate cancer.

## INTRODUCTION

Prostate cancer is the second most commonly diagnosed cancer in males worldwide and, in the United States, the most commonly diagnosed invasive cancer in males. Prostate cancer is also the fifth leading cancer-related cause of death worldwide (1). The increase in life expectancy will lead to an increase in disease incidence, which is becoming an epidemic in terms of male public health. In the United States, prostate cancer is also the second most common type of cancer and the second leading cause of cancer death (second only to lung cancer). In 2017, the incidence and deaths from this disease were 161.360 cases and 26.730 cases, respectively (2).

As the presence and location of the primary or recurrent tumors are critical for planning patient management, a vast range of imaging modalities are being assessed as tools for the evaluation of patients with prostate cancer in primary and secondary staging (1, 2).

While the introduction of PSA-screening has led to earlier diagnosis of prostate cancer, a subset of patients developed high-risk of metastatic disease (3). A more accurate alternative for assessing disease staging is crucial for treatment decisions. However, all current conventional imaging modalities show limitations, and optimizing these imaging modalities is an intense and rapidly developing field of research. In the last decades, we have seen the development and improvement of functional imaging. Among those, combined positron emission tomography (PET)/computed tomography (CT) is one of the most promising techniques (4).

^68^Ga-PSMA PET-CT is a non-invasive diagnostic tool to evaluate prostate cancer with increased prostate-specific membrane antigen (PSMA) expression. PSMA is a protein expressed on dysplastic prostate cells, with levels of expression of 100-1000 times that of healthy cells, which increase even further with higher stages and grades of prostate cancer (5, 6).

^68^Ga is a positron emitter obtained from a ^68^Ge/^68^Ga generator system with 68min of half-life. PSMA demonstrated to have high affinity and specific internalization into prostate cancer cells (7). ^68^Ga-PSMA-11 was first synthesized and evaluated by the Heidelberg group in Germany, and its accumulation is proportional to the expression level of PSMA (8). Other PSMA ligands, such as ^68^Ga-PSMA-617 and ^68^Ga-PSMA-I&T, showed similar distribution and image properties to ^68^Ga-PSMA-11 (6, 8), the reason why all three are known as ^68^Ga-PSMA.

To date, the use of ^68^Ga-PSMA has been well reported, and initial staging revealed superior sensitivity and specificity profiles compared to conventional choline-based tracers (7). Not only diagnosis but also among the management changes observed in the studies, the proportion of inter and intra-modality was relatively similar, indicating that ^68^Ga-PSMA-PET may help better plan the optimal dose, site, and volume of radiation in the case of salvage radiotherapy. We systematically reviewed the literature outlining the use of ^68^Ga-PSMA PET imaging in prostate cancer. Our primary objective was to perform a literature review to determine ^68^Ga-PSMA PET accuracy in prostate cancer, and our secondary objective was to identify predictors of positive ^68^Ga-PSMA PET.

## MATERIALS AND METHODS

### Bibliographic search

A systematic review was performed under the Cochrane Collaboration and Preferred Reporting Items for Systematic Review and Meta-analysis (PRISMA) guidelines (9, 10).

We performed a comprehensive literature search of PubMed, Cochrane Library, Embase, Web of Science, Scielo, Scopus and Lilacs database, without date restriction up to 05/04/2019, using the following MeSH vocabulary key words and free text words: ((Prostate Neoplasms OR Prostate Neoplasm OR Prostatic Neoplasm OR Prostate Cancer OR Prostate Cancers OR Prostatic Cancer OR Prostatic Cancers) AND (Positron Emission Tomography OR PET) AND (PROSTATIC SPECIFIC MEMBRANE ANTIGEN OR PSMA OR GALLIUM OR GA)).

### Inclusion and exclusion criteria

Patients: patients diagnosed with prostate cancer and patients initially submitted to radical prostatectomy (RP) or radiotherapy with curative intent who showed biochemical recurrence defined as a prostate-specific antigen (PSA) elevation of ≥0.2ng/mL in patients with primary prostatectomy or as a PSA above the nadir after primary radiation therapy (according to the ASTRO 1996 criteria (11)).

Index test: ^68^Ga-PSMA-11 PET

Target condition: Diagnostic, staging, local recurrence, lymph nodal spread, distant metastasis.

Study design: Diagnostic accuracy cross-sectional study with prospective and retrospective approaches.

Exclusion criteria: case reports, animal, and phantom studies were excluded.

No language or sample-size restrictions were used.

### Reference standard

A composite standard including changing in PSA values, clinical follow-up, and histopathological findings.

### Outcome measures

The outcome measures included identification of predictors of ^68^Ga-PSMA-PET positivity, sensitivity, specificity, positive predictive value (PPV), negative predictive value (NPV), and overall accuracy.

### Study selection

The titles and abstracts retrieved from bibliographic searches were independently screened by two authors (MCS and PDB). The full texts of all relevant articles were obtained and independently assessed for inclusion by the same authors aforementioned. Studies that did not fulfill the inclusion criteria were excluded.

### Quality assessment

The studies were independently assessed by two authors (MCS and PDB) using the Quality Assessment of Diagnostic Accuracy Studies-2 (QUADAS 2) checklist tool (12). The QUADAS-2 tool assesses four domains: risk of bias in patient selection, index test, reference standard, and the timing of reference standard. Each paper was scored independently by two evaluators (MCS and PDB), and discrepancies were discussed and resolved in common agreement.

### Data extraction

The following information was extracted from each study: sample size, age of the patients, indication for PET (diagnosis, primary staging or recurrent disease staging), PSA level, previous therapies, initial cancer stage, ^68^Ga-PSMA-11 PET characteristics, rates of positive PET, and pathology correlation data (when available). When pathology correlation data were available, numbers of true positives, false positives, true negatives and false negatives were collected as appropriated. The ^68^Ga-PSMA-PET for diagnosis, primary staging, and recurrent cancer staging purposes data were displayed separately, when available.

The extracted data were collected in Excel 2007 (Microsoft Corporation, Redmond, CA, USA), and analysis was performed using Meta-Disc 1,4 (13). The detection rates were pooled using the generic inverse variance approach in the random-effects model (14). Heterogeneity in the meta-analysis of detection rates was assessed using the X^2^ statistic in the I^2^ statistic (10). The I^2^ statistic indicates the percentage of the overall variability that can be attributed to between-study (or inter-study) variability, as opposed to within-study (or intra-study) variability. An I^2^ more significant than 50% is considered to indicate substantial heterogeneity (10).

We explored the variability in diagnostic accuracy across studies by plotting the estimates of the observed sensitivities and specificities in forest plots and receiver-operating characteristic (ROC) space. Whenever data for computing true-positive, false-negative, true-negative, and false-positive rates were available, we performed meta-analyses using the bivariate model to produce summary sensitivities and specificities (13). We remade the calculations, excluding one article at a time, to verify if one of the studies was the main responsible for the heterogeneity in the meta-analyses. If substantial heterogeneity in the prevalence among the studies was observed, we estimated PPVs and NPVs by considering their relationship in the sensitivity, specificity and prevalence, and generating different scenarios with different prevalence. The significance level was set at **α**=0.05.

In patients undergoing a scan for disease recurrence, we correlated the data (sensitivity and specificity) to PSA level, in subgroups divided as follow: PSA level <0.5ng/mL, PSA level between 0.5ng/mL and 1ng/mL, PSA level between 1ng/mL and 2ng/mL and PSA level higher than 2ng/mL.

## RESULTS

### Identification of Studies

**Figure 1:**
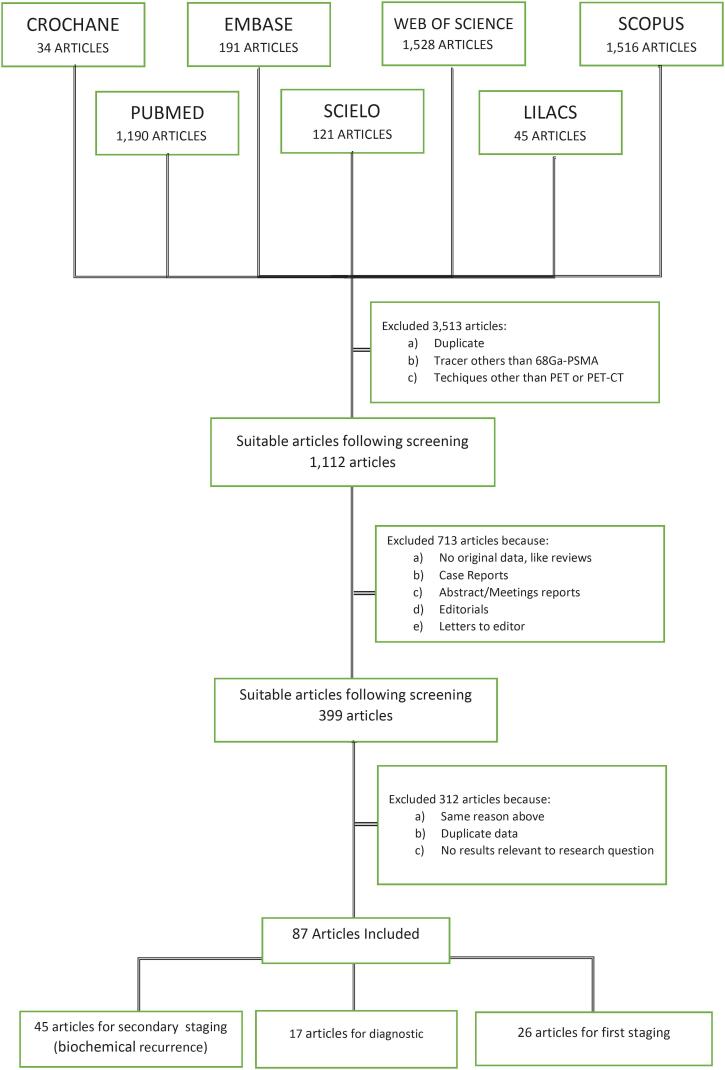
Summary of the study selection process.

Figure-1 summarizes the process of identification and selection of studies. The electronic search was complemented by manually checking the reference lists in review papers and all included studies. Overall, we included 87 studies comprising of a total of 9046 patients (range: 6-635 patients per study): 17 studies on diagnostic (15-31), 26 studies on staging (32-57), and 45 studies on restaging (5, 23, 57-100).

### Study design and quality

A total of 52 studies used a retrospective design (6, 15, 21-28, 31-34, 37-41, 43, 45, 46, 48, 51-56, 58-64, 67, 72-77, 79, 80, 82, 85, 92, 93, 96-98), 27 studies used a prospective design (18, 19, 29, 30, 35, 36, 44, 47, 49, 57, 66, 68, 70, 71, 78, 81, 83, 84, 87-91, 94, 95, 99, 100), and one of them used cohort design (65). Seven studies did not indicate how data were included or obtained (16, 17, 20, 42, 50, 69, 86).

While the patient selection was generally acceptable in most of the studies included, a few studies did not report the inclusion criteria clearly. All the studies reported methodology for the index test with clarity and were, thus, not considered a significant source of potential bias. There was a broad variability in the reference test: Prostate biopsy results were used in ten studies (19, 21, 23, 32-35, 39, 54, 83), histologic results from radical prostatectomy or lymphadenectomy in 35 studies (6, 15, 16, 18, 20, 22, 24, 25, 27, 29, 31, 34, 36, 38, 41, 46, 48, 49, 52, 54, 55, 59, 62, 68, 77, 78, 83, 84, 86, 87, 90, 92, 95-97) and combined outcomes using additional imaging with magnetic resonance imaging (MRI) or bone scan and/or clinical follow-up and/or response to treatment (mostly PSA testing courses over time) in 45 studies (17, 26, 28, 30, 37, 40, 47, 50, 51, 53, 56-58, 60, 61, 63-67, 69-76, 79-82, 85, 88, 89, 91, 93, 94, 98-100) (Tables 1 a-c).[Table t1b]

**Table 1a:** Summary of contents of study design – Diagnosis.

Author	*D	Country	N	Scan Time	Reference text	Age	Patient Characteristics	Results
Eiber, et al. (15)	R	Germany	53	60	Histology	62-72	Confirmed Prostate Cancer	68Ga-PSMA PET improves accuracy for diagnosis and localization of prostate cancer
Budäus, et al. (27)	R	Germany	30	–	Histology	44-75	Confirmed Prostate Cancer	68Ga-PSMA PET improves accuracy for diagnosis and localization of prostate cancer. The accuracy depends on the size of lymphnodes
Gupta, et al. (25)	R	India	11	–	Histology	46-46	High risk prostate cancer	68Ga-PSMA PET improves accuracy for diagnosis and localization of prostate cancer
Hicks, et al. (28)	R	USA	32	71±14’	MRI	62-71	High risk prostate cancer	68Ga-PSMA PET sensitivity is better than MRI
Lopci, et al.(29)	P	Italy	45	60’	Histology	64.52	High risk prostate cancer	68Ga-PSMA PET is indicated to diagnose Prostate cancer when MRI is negative or contraindicated
Sanli, et al. (26)	R	Turkey	109	45-60’	PSA level, Gleason score	48-79	Confirmed Prostate Cancer	68Ga-PSMA PET improves accuracy for diagnosis and localization of prostate cancer
Wu, et al. (30)	P	USA	45	50-100’	Radiotherapy response	66-74	Confirmed Prostate Cancer	68Ga-PSMA PET changed radiotherapy area in 53%
Zhang, et al. (31)	R	China	58	601	Histology	55-85	High risk prostate cancer	68Ga-PSMA PET prostate cancer detection is superior than nanogram
Zamboglou, et al. (16)	–	Germany	7	–	Histology	52-74	Patients with prostate cancer candidates with subsequent prostatectomy	68Ga-PSMA PET and MRI have high sensitivity and specificity, the combination of techniques improves performance
Sachpekidis, et al. (17)	–	Germany	24	60	PSA	43-84	Confirmed Prostate Cancer	68Ga-PSMA PET has a high detection rate
Fendler, et al. (18)	P	Germany	21	60	Histopathology	–	Confirmed Prostate Cancer	68Ga-PSMA PET detects the location and extent of primary prostate cancer
Zamboglou, et al. (20)	–	Germany	9	60	Histology	49-74	Confirmed Prostate Cancer	68Ga-PSMA PET had a good correlation with histology
Sahlmann et al. (21)	R	Germany	35	60-180	Biopsy or unequivocal CT findings	49-78	Confirmed Prostate Cancer	68-Ga PSMA PET is a tool that can be used to diagnose metastases of prostate cancer
Rahbar, et al. (22)	R	Germany	6	65	Histopathology	56-73	High risk prostate cancer	68Ga-PSMA PET can detect injuries and collaborate on clinical strategy
Hijazi, et al. (23)	R	Germany	35	60-180	PSA and biopsy	49-77	Confirmed Prostate Cancer or Patients with biochemical relapse	68Ga-PSMA PET improves the accuracy of micro-metastases diagnosis
Kabasakal, et al. (24)	R	Turkey	28	5-60	Histopathology	55-85	Confirmed Prostate Cancer	68Ga-PSMA PET is valuable for assessing primary prostate cancer
Rhee, et al. (19)	P	Australia	20	90	Biopsy	62±7	Confirmed Prostate Cancer	Both PSMA and MRI failed to diagnose an expressive number of lesions

* **D** = Study design; **R** = for retrospective study; **P** = prospective study; **C** = cohort

**Table 1b t1b:** Summary of contents of study design – Staging.

Author	*D	Country	N	Scan Time	Reference text	Age	Patient Characteristics	Results
Uprimny, et al. (33)	R	Austria	90	60	Biopsy/Gleason score	47-83	Patients with untreated prostate cancer	68Ga-PSMA PET is correlated with PSA and Gleason score
Afshar-Oromieh, et al. (43)	–	Germany	112	60-180’	CT or MRI	69.8±7.9	Suspected prostate cancer	PET/CT with 68Ga-PSMA-11 at 3 h after injection is a valuable method to clarify unclear findings of regular scans conducted at 1 h after injection or to find new PCa lesions because most PCa lesions present with a higher uptake and contrast in late scans.
Bräuer, et al. (41)	R	Germany	27	NA	change in therapeutic management	67.8±8.2	Patients referred for 223Ra-dichloride therapy	Additional 68Ga-PSMA-PET as a gatekeeper between conventional staging and 223Ra-dichloride therapy can provide valuable additional information with regard to visceral metastases and tumour manifestations without adequate bone mineral turnover.
Demirkol, et al. (44)	R	Turkey	22	NA	PSA levels, treatment response	43-86	Suspected Prostate cancer	PSMA based nuclear imaging has significantly impacted our way of handling patients with prostate cancer.
Dyrberg, et al. (45)	P	Denmark	55	NA	NaF-PET/CT, WB-MRI	54-91	patients with biopsy-proven prostate cancer referred by the clinicians for the standard bone imaging method at our institution, NaF-PET/CT	The overall accuracy of PSMA-PET/CT was significantly more advantageous compared to WB-MRI, but not to NaF-PET/CT.
Ergül, et al. (46)	R	Turkey	78	60’	prostate-specific antigen (PSA) values, Gleason score (GS), and d'Amico risk classification.	47-88	newly diagnosed prostate carcinoma and no previous therapy	PSMA PET/CT is well suited for detecting the intraprostatic malignant lesion in patients with newly diagnosed prostate cancer.
Grubmüller, et al. (47)	R	Austria	117	60’	PSA levels	68-76	hormone-naïve BCR patients	They confirm the high performance of PSMA-PET imaging for the detection of disease recurrence sites in patients with BCR after RP, even at relatively low PSA levels
Hruby, et al. (48)	P	Australia	109	Min. 45'r	conventional imaging with bone scan, CT and multiparametric MRI	56-86	Men prior to definitive EBRT for intermediate and high-risk prostate cancer	PSMA-PET/CT identified the primary in 99% of patients, and altered staging in 21% of men with intermediate or high-risk prostate cancer referred for definitive EBRT compared to CT, bone scan and multiparametric MRI.
Kuten, et al. (49)	R	Israel	137	–	PSA levels, clinical history, imaging reports and histopathological reports	68.5±6.8	newly diagnosed intermediate- and high-risk prostate cancer were referred for initial staging	Ga-PSMA PET/CT shows promise as a sole whole-body imaging modality for assessing the presence of soft tissue and bone metastases in the setting of prostate cancer.
Lengana, et al. (50)	P	South Africa	113	60’	bone scan	43-88	patients with biopsy-proven prostate cance	8Ga-PSMA PET/CT is superior to and can potentially replace bone scan in the evaluation for skeletal metastases
Roach, et al. (58)	P	Australia	431	45-60’	follow-up questionnaire	68.5±8.0	Patient in primary staging of intermediate- and high-risk prostate cancer	68Ga-PSMA PET/CT scans detect previously unsuspected disease and may influence planned clinical management in a high proportion of patients with prostate cancer.
Soldatov, et al. (51)	–	Germany	108	NA	PSA level	68.7±7.3	Patients with increased prostate-specific antigen levels	SMA-ligand PET/CT-guided RT for relapsed PCa with limited tumor burden allowed individualization of treatment approaches, provided effective local control, and resulted in considerably prolonged biochemical progression-free survival
Thomas, et al. (52)	R	Germany/ USA	21	80’	conventional plans	53-84	prostate cancer patients without previous local therapy	the use of 68Ga-PSMA-PET/CT image information allows for more individualized prostate treatment planning
Uprimny, et al. (53)	R	Austria	203	60'or until 5 min	PET scans 60 min p.i	54-92	PC patients with biochemical failure	Performance of early imaging in 68Ga-PSMA-11 PET/CT in addition to whole-body scans 60 min p.i. increases the detection rate of local recurrence in PC patients with biochemical recurrence
Uprimny, et al. (54)	R	Austria	16	40-70 sec	18F-NaF PET	59-82	metastatic PC patients with known skeletal metastases	n comparison to 68Ga-PSMA-11 PET/CT, 18F-NaF PET/CT detects a higher number of pathologic bone lesions in advanced stage PC patients scheduled for radionuclide therapy.
Uslu-Besli, et al. (55)	R	Turkey	28	45’	Bone scan	67.3±7.4	prostate cancer patients	Ga-68 PSMA PET/CT has higher sensitivity, specificity, and accuracy compared to bone scan in terms of bone metastasis detection in prostate cancer patients
Vinsensia, et al. (42)	R	Germany	147	60±10	conventional CT	44-87	prostate cancer patients	68Ga-PSMA PET is a promising modality in biochemical recurrent prostate cancer patients for N staging. Conventional imaging underestimates LN involvement compared with PSMA molecular staging score in each GS cohort.
von Klot, et al. (56)	R	Germany	21	NA	Histology	68.1±6.0	patients with prostate cancer before either open or laparoscopic radical prostatectomy	68Ga-PSMA I&T PET/CT prior to radical prostatectomy can contribute to presurgical local staging of prostate cancer.
Wong, et al. (57)	R	Australia	131	NA	impacted on management	49-84	patients with newly diagnosed prostate cancer prior to consideration of definitive treatment were included in this study.	The use of 68 Ga-PSMA PET scans in initial staging can have a significant impact on staging and management when compared to current conventional imaging modalities.
Fendler, et al. (35)	R	Multicentric	50	54	Histology, biopsy	49-83	Confirmed Prostate Cancer	Interobserver correlation for staging is good when observers have medium or large experience
Zang, et al. (36)	P	China	40	60	biopsy	53-91	Patients with biochemical recurrence of prostate cancer	68GA-PSMA PET has high sensitivity and specificity
van Leeuwen, et al. (37)	P	Australia	30	60	Histopathology	65.0±8.5	Patients at Risk for Prostate Cancer	68Ga-PSMA PET has high specificity and moderate sensitivity in seven lymph node metastases
Pyka, et al. (38)	R	Germany	126	60	"best valuable comparator " (Clinical and Imaging Information)	68.9±7.7	Confirmed Prostate Cancer	PSMA is better than bone scintigraphy in both staging and detection
Herlemann et al. (39)	R	Germany	34	60	Histopathology	50-80	Confirmed Prostate Cancer	68-PSMA PET is accurate in detecting the spread of the tumor
Giesel et al. (40)	R	Germany	10	60	Biopsy	61-74	Confirmed Prostate Cancer	MP-MRI and PSMA-PET / CT are accurate in localizing prostate cancer
Maurer et al. (34)	R	Germany	130	59	Biopsy	45-84	Patients with suspected prostate cancer	68Ga-PSMA PET has superior performance in detecting lymph node stage

* **D** = Study design; **R** = retrospective study; **P** = prospective study; **C** = cohort.

**Table 1c:** Summary of contents of study design – Recurrence.

Author	D*	Country	N	Scan Time	Reference text	Age	Patient Characteristics	Results
Schmuck, et al. (60)	R	Germany	240	180	PSA, Histology and follow-up	59.8±7.5	Patients with recurrent prostate cancer	68Ga-PSMA PET had high detection rates in patients with persistent PSA or biochemical persistence of prostate cancer.
Uprimny, et al. (61)	R	Austria	80	60	PSA	47-92	Patients with recurrent prostate cancer	68Ga-PSMA PET is reliable in distinguishing lesion from bladder activity
Berliner, et al. (62)	R	Germany	83	80	PSA	68±7	Patients with biochemical recurrence of prostate cancer	68Ga-PSMA PET has high rates of detection of recurrent cancer, the higher the level of PSA the greater the detection rate
Einspieler, et al. (63)	R	Germany	118	60	Histology	50-87	Patients with biochemical recurrence of prostate cancer	68Ga-PSMA PET has high levels of cancer detection
Kabasakal, et al. (64)	R	Turkey	50	60	Change in PSA levels posterior 171Lu-PSMA treatment	67.3±8.7	Patients referred for restaging	68Ga-PSMA PET is accurate to reestablish patients with relapse
Schwenck, et al. (65)	R	Germany	123	60	PSA	–	Patients with recurrent prostate cancer	PSMA is more accurate than C-choline to detect lymph nodes and bone lesions
Hruby, et al. (66)	C	Australia	419		Biochemical recurrence	–	Patients submitted to radiotherapy	68Ga-PSMA PET detected all cases of biochemical recurrence
Dietlein, et al. (87)	P	Germany	129	60-120	PSA	–	Patients with biochemical recurrence of prostate cancer	F-DCFPyL is not inferior than 68Ga-PSMA-HBED-CC
Meredith, et al. (68)	R	Australia	532	60	PSA	44-85	Patients with biochemical recurrence of prostate cancer	68Ga-PSMA PET s a new imaging modality to detect prostate cancer
Bluemel, et al. (59)	R	Germany	32	60	PSA	69.4±6.8	Patients with biochemical recurrence of prostate cancer and negative Choline-PET/CT	Including 68Ga-PSMA PET in Choline-PET / CT negative patients increases the ratio of positive diagnoses
Pfister, et al. (69)	P	Germany	28	45	Histopathology	46-79	Patients with biochemical recurrence of prostate cancer	68Ga-PSMA PET is more accurate than Fluoroethylcholine PET/CT
Sachpekidis, et al. (70)	–	Germany	31	60	PSA	54-77	Patients with biochemical recurrence of prostate cancer	68Ga-PSMA PET has promising and satisfactory detection levels
Henkenberen, et al. (71)	P	Germany	29		PSA	51-86	Patients with biochemical recurrence of prostate cancer	Radiation therapy guided by 68Ga-PSMA PET has good results
van Leeuwen, et al. (72)	P	Australia	70	45	PSA	57-67	Patients with biochemical recurrence of prostate cancer	68Ga-PSMA PET has reasonable performance in identifying recurrence of prostate cancer in patients with low PSA
Verburg, et al. (73)	R	Germany	155	60	PSA	43-86	Patients with biochemical recurrence of prostate cancer	68Ga-PSMA PET is a promising and clinically useful tool
Ceci, et al. (74)	R	Austria	70	60	PSA	38-91	Patients with biochemical recurrence of prostate cancer	68Ga-PSMA PET has the potential confirmed in patients with biochemical relapse
Eiber, et al. (75)	R	Germany	248	54	PSA	46-85	Patients with recurrent prostate cancer	68Ga-PSMA PET has a substantially higher detection rate compared to other imaging tests
Afshar-Oromieh, et al. (78)	R	Germany	310	60	PSA and Histology	46-86	Patients with recurrent prostate cancer	68Ga-PSMA PET can detect the recurrence of prostate cancer in a large number of patients
Afshar-Oromieh, et al. (6)	R	Germany	37	60	PSA and Histology	57-85	Patients with recurrent prostate cancer	68Ga-PSMA PET identifies a larger number of patients than Fluoroethylcholine
Afaq, et al. (77)	R	UK	100	60	Gleason grade, stage, presence of metastatic disease, PSA velocity, or PSA doubling time	79-89	patients with BCR	68Ga-PSMA PET/CT altered management in 39% of patients with BCR, and changes occurred more often in patients with radical radiotherapy treatment, positive 68Ga-PSMA scan results, and higher PSA levels.
Akdemir, et al. (79)	P	Turkey	121	60	Conventional imaging	51-92	Patients with recurrent prostate cancer	68Ga-PSMA-PET/CT is useful for re-staging patients with RPCa and has improved performance compared with CI for disease detection.
Barbaud, et al. (80)	R	France	42	60	FCH PET-CT	57-80	Prostate Cancer patients with previously negative or doubtful 18F-Choline (FCH)	Performing a PSMA PET-CT when FCH PET-CT was doubtful or negative allows the recurrence localization in more 80% of patients and this had a major clinical impact, as it resulted in treatment change in more than 70% of patients as well as a significant decrease in PSA levels in more than 60% of them.
Bashir, et al. (81)	R	UK	28	60	Follow up	50-76	patients with early BCR post RP	Our findings show that PSMA-PET/CT has a high detection rate in the eBCR setting following RP, with a large proportion of patients found to have fewer than three lesions.
Calais, et al. (82)	P	USA	101	52-96	Referring physicians questionnaires	–	patients with proven prostate adenocarcinoma and BCR after prostatectomy or definitive radiotherapy.	This prospective referring physician–based survey shows a significant impact (54/101; 53%) of 68Ga-PSMA-11 PET/CT on the actual management of prostate cancer patients with BCR. Importantly, intended management changes after 68Ga-PSMA-11 PET/CT were further modified in almost 50% of the patients, underlining the limitations of survey-based management assessment
Calais, et al. (83)	R	USA	270	59	Referring physicians questionnaires	43-90	patients with prostate cancer BCR	Information from 68Ga-PSMA-11 PET/CT brings about management changes in more than 50% of prostate cancer patients with BCR (54/101; 53%). However, intended management changes early after 68Ga-PSMA-11 PET/CT frequently differ from implemented management changes.
Caroli, et al. (84)	P	Italy	314	50	PSA level	44-92	Patients with BCR of PCa after radical surgery and/or radiotherapy with or without androgen-deprivation therapy	We confirmed the value of 68Ga-PSMA PET/CT in restaging PCa patients with BCR, highlighting its superior performance and safety compared with choline PET/CT. Higher PSApet was associated with a higher relapse detection rate.
Ceci, et al. (85)	P	Italy	332	60	PSA level	54-83	Men with proven PCa, submitted surgery or radiotherapy as definitive therapy, with proven BCR, prostate-specific antigen (PSA) 0.2-2 ng/mL, ≥ 35 years	Our data confirmed the efficacy of 68Ga-PSMA-11 PET/CT for detecting local vs systemic disease in PCa patients presenting PSA failure after radical therapy. Furthermore, 68Ga-PSMA-11 PET/CT detection rate is different depending on the clinical stage of BCR, and this information should be taken into consideration by referring physicians.
Dewes, et al. (86)	R	Germany	15	NA	PSA levels	59-82	patients treated for PC between August 2013 and April 2015 that were ere planned for definitive RT of the prostate with treatment planning based on CT, MRI and 68Ga-PSMA-PET-imaging.	The integration of (68)Ga-PSMA-PET-imaging into the RT treatment planning process can be useful for detailed target volume planning. The performance of a (68)Ga-PSMA-PET frequently leads to changes in the TNM stage, altering the RT treatment regimen and the target volume. A prospective trial is underway to evaluate the impact of (68)Ga-PSMA-PET based treatment planning on outcome.
Dietlein, et al. (67)	P	Germany	129	60-120	18F-DCFPyL	–	patients with biochemical recurrence of prostate cancer	Our data suggest that 18F-DCFPyL is noninferior to 68Ga-PSMA-HBED-CC, while offering the advantages of 18F labeling. Our results indicate that imaging with 18F-DCFPyL may even exhibit improved sensitivity in localizing relapsed tumors after prostatectomy for moderately increased PSA levels. Although the standard acquisition protocols, used for 18F-DCFPyL and 68Ga-PSMA-HBED-CC in this study, stipulate different activity doses and tracer uptake times after injection, our findings provide a promising rationale for validation of 18F-DCFPyL in future prospective trials.
Fendler, et al. (88)	P	USA	635	64+	Histopathologic analysis	44-95	patients with biochemically recurrent prostate cancer after prostatectomy	Using blinded reads and independent lesion validation, we establish high PPV for 68Ga-PSMA-11 PET, detection rate and interreader agreement for localization of recurrent prostate cancer.
Fennessy, et al. (89)	P	Australia	62	60	PSA Level	47-89	men with prostate cancer undergoing clinically indicated 68 Ga-PSMA PET/CT	Intravenous Frusemide given with 68 Ga-PSMA reduces excretion artefact, and improves diagnostic certainty. Frusemide should be considered for all 68 Ga-PSMA PET/CT imaging protocols.
Gauthé, et al. (90)	P	France	33	60	PSA level	55-79	patients presenting biochemical recurrence of prostate cancer whose 18Ffluorocholine (FCH) PET/CT was non-contributive	68Ga-PSMA-11 PET/CT is useful in detecting recurrence of prostate cancer, by identifying residual disease which was not detected on other imaging modalities and by changing management of 2 patients out of 3.
Hamed, et al. (91)	P	Egypt	188	60	Histopathology, clinical and imaging follow up	56-79	patients who exhibited rising of PSA level on a routine follow-up examination after definitive treatment of PC	68Ga-PSMA PET/CT is a valuable tool for the detection of PC local recurrence or extraprostatic metastases following risin
Hijazi, et al. (24)	R	Germany	35	60-180	PSA and biopsy	49-77	Confirmed Prostate Cancer or Patients with biochemical relapse	68Ga-PSMA PET improves the accuracy of micro-metastases diagnosis
Hope, et al. (92)	P	USA	150	63±10	Referring physicians questionnaires	69±6.9	a prostate-specific antigen (PSA) doubling time of less than 12 mo after initial treatment	68Ga-PSMA-11 PET resulted in a major change in management in 53% of patients with biochemical recurrence. Further studies are warranted to investigate whether PSMA-based management strategies result in improved outcomes for patients.
Jilg, et al. (93)	R	Germany	30	50	Imunohistochemistry	–	patients with the suspicion of exclusively nodal PCa-relapse after primary therapy underwent a template pelvic and/or retroperitoneal salvage-LND after whole body 68-Ga-PSMA-PET/CT	In men with biochemical PCa-relapse and positive PSMA-PET/CT, PET/CT detects metastatic affected anatomical regions with high accuracy at a main region and at a subregion-level. If the decision for salvage-LND is prompted by a positive PSMA-PET/CT, the size of metastases is crucial for accurate detection of affected regions.
Mattiolli, et al. (94)	R	Brazil	126	60	PSA levels	43-89	prostate cancer patients submitted to the 68Ga-PSMA PET / CT due to biochemical recurrence	68Ga-PSMA PET / CT in prostate cancer patients with biochemical recurrence has a high impact in patient management.
McCarthy, et al. (95)	P	Australia	238	60	PSA levels	46-91	All patients had histologically confirmed PCa with biochemical relapse after definitive prostatectomy or radiation therapy. Patients with biochemical relapse at least 6 months after previous response to systemic (hormonal) treatment were also included.	PSMA PET/CT is significantly more sensitive than standard restaging imaging, and it may be useful in identifying patients for subsequent targeted therapy.
Morigi, et al. (96)	P	Australia	38	45	8F-fluoromethylcholine	54-81	A sample of men with a rising PSA level after treatment, eligible for targeted treatment, was prospectively included	In patients with biochemical failure and a low PSA level, (68)Ga-PSMA demonstrated a significantly higher detection rate than (18)F-fluoromethylcholine and a high overall impact on management.
Pfister, et al. (69)	P	Germany	28	45	Lymphadenectomy	46-79	Prostate Cancer patients who underwent sLAD after PET/CT	In the present series Ga-PSMA PET/CT shows a better performance than FEC PET/CT with a significantly higher NPV and accuracy for the detection of locoregional recurrent and/or metastatic lesions prior to salvage lymphadenectomy.
Rauscher, et al. (97)	R	Germany	48	49-63	Salvage lymphadenectomy	66-74	patients with biochemical recurrence who underwent 68Ga-prostate-specific membrane antigen (PSMA) HBED-CC PET/CT or PET/MR and salvage lymphadenectomy	68Ga-PSMA HBED-CC PET imaging is a promising method for early detection of LNM in patients with biochemical recurrent prostate cancer. It is more accurate than morphologic imaging and thus might represent a valuable tool for guiding salvage lymphadenectomy.
Schmidkonz, et al. (98)	R	Germany	93	180-240	Follow up	51-81	subjects conforming to the following criteria: histopathologically confirmed PC by needle biopsy and no primary therapy	MIP-1404 SPECT/CT has a high accuracy and low interobserver variability in the diagnosis of PC and allows detection of lymph node and bone metastases in a significant proportion of as yet untreated PC patients.
Schmidt-Hegemann, et al. (76)	R	Germany	172	60	Conventional CT	46-86	Prostate Cancer patients underwent 68Ga-PSMA PET/CT before radiotherapy	Compared with conventional CT, 68Ga-PSMA PET/CT had a remarkable impact on radiotherapeutic approach, especially in postoperative patients.
Walacides, et al. (99)	R	Germany	25	?	Conventional Imaging	50-85	patients with biochemical prostate cancer recurrence after primary prostatectomy underwent 68 Ga-PSMA ligand PET/CT in addition to conventional imaging techniques such as CT and/or MR imaging for restaging	68 Ga-PSMA ligand PET/CT is superior to conventional cross-sectional imaging for the delineation of lymph node metastases from prostate cancer.
Wondergem, et al. (100)	P	Netherlands	65	60 or 120	PSA level	52-84	prostate cancer patients who were referred to our nuclear medicine department for 18F-DCFPyL PET/CT were included	18F-DCFPyL PET/CT images at 120 min after injection yield a higher detection rate of prostate cancer characteristic lesions than images at 60 min after injection. Further studies are needed to elucidate the best imaging time point for 18F-DCFPyL.
Zacho, et al. (101)	P	?	68	60	18F-NaF PET/CT	47-80	PCa patients with BCR	68Ga-PSMA PET/CT and 18F-NaF PET/CT showed comparable and high diagnostic accuracies for detecting bone metastases in PCa patients with BCR.

* **D** = Study design. R for retrospective study, **P** = prospective study, **C** = cohort.

### Pooled sensitivity, specificity, and diagnostic odds ratios; likelihood ratios

**Figure 2:**
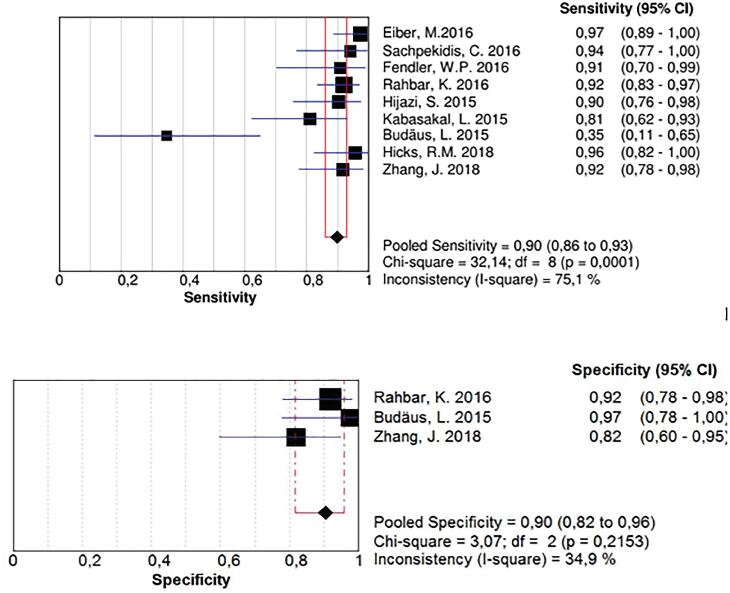
Pooled sensibility and specificity in diagnosis scenario, considering only studies which included possible non-cancer cases (false positive).

The availability of original data and the possibility of constructing a two-by-two table were minimum requirements (6, 15, 17, 18, 22-24, 32, 33, 36, 37, 39-49). We excluded studies that did not provide us with enough data in terms of sensitivity, specificity, diagnostic odds ratios, and likelihood ratios analyses to create a 2X2 Table. Overall, 34 studies met the criteria for this meta-analysis, comprehending a total of 4532 patients submitted to ^68^Ga-PSMA PET; among those, nine studies (287 patients) in diagnostic setting and four studies for staging purposes, and 22 studies for restaging, in a total of 4050 patients (note that 1 study included staging and restaging patients). For diagnosis, the sensitivity was 0.90 (0.86 to 0.93), with an inconsistency of 75.1% and a specificity of 0.90 (0.82 to 0.96), not considering case-only confirmed patients (Figure-2).

**Figure 3:**
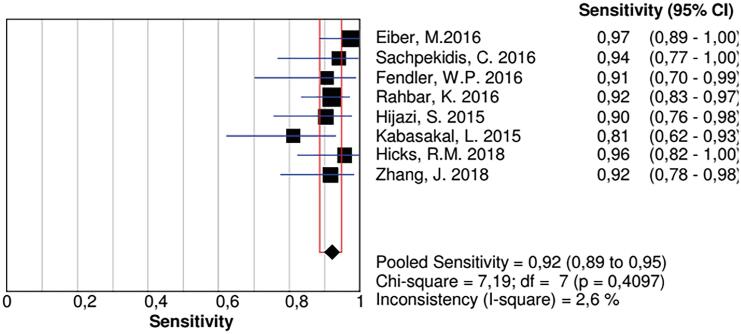
A secondary analysis, when Budaus, L. Study was withdraw from the pool, the inconsistency became much lower, not changing, substantially, the sensitivity of the method.

We carried out a secondary analysis, withdrawing studies, one by one, in order to identify which study generated such heterogeneity. Recalling Budaus, L 2015 study, we obtained a pooled sensitivity of 0.92 (0.89 to 0.95) with 2.6% heterogeneity (Figure-3).

**Figure 4:**
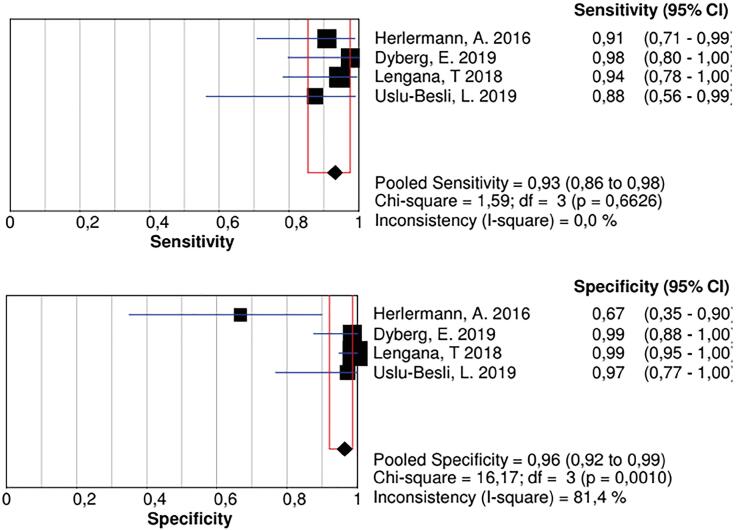
Sensitivity and Specificity analysis in staging prostate cancer patients. Note in specificity analysis the high inconsistency among these studies.

**Figure 5:**
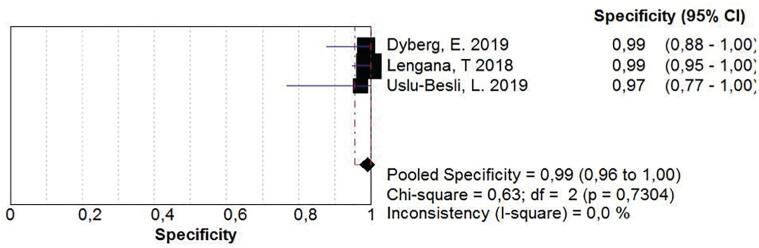
Specificity analysis in staging prostate cancer patients, after secondary analysis that showed Herlermann, A. study was responsible for the great inconsistency. Recalling the study, the specificity was 0.99 (0.96 to 1.00) with no inconsistency between the studies.

**Figure 6:**
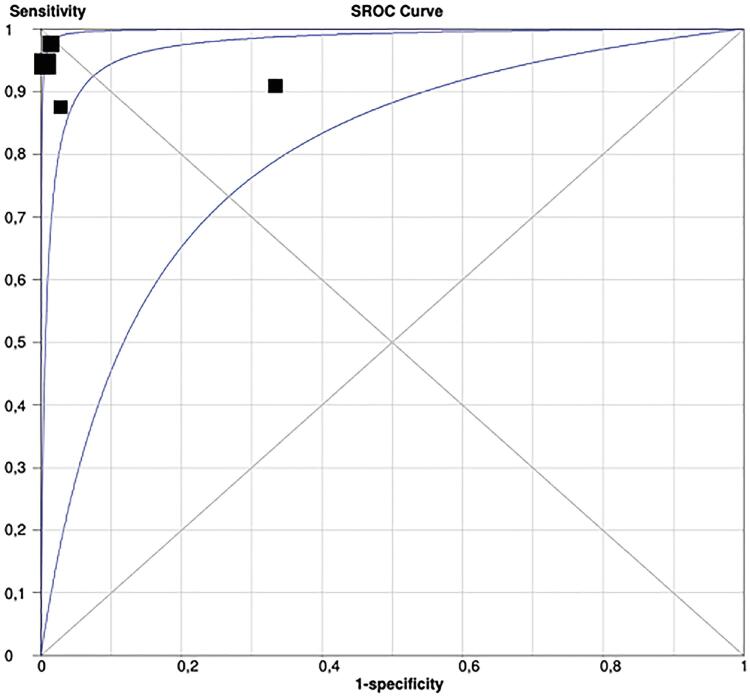
ROC curve for 68Ga-PSMA-PET in diagnosis setting, showing an area under the curve of 0.9731.

The group of studies that enrolled patients for staging indication, resulted in a sensitivity of 0.93 (0.86-0.98), with a low inconsistency, and a specificity of 0.96 (0.92-0.99), but with a significant inconsistency (Figure-4). After a secondary analysis, when Herlermann, the study found to be responsible for the inconsistency, was withdrawn from the pool, the specificity was 0.99 (0.96-1.00) (Figure-5). A summary ROC (sROC) curve confirmed the high value for this imaging modality in the staging setting with an area under the curve of 0.9731 (Figure-6).

**Figure 7:**
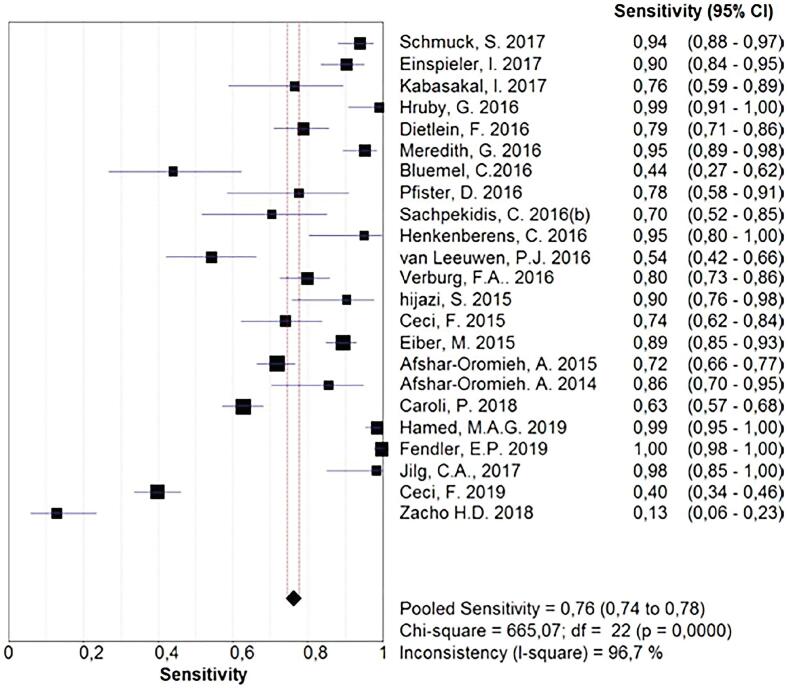
68Ga-PSMA-PET sensitivity for restating patients with biochemical recurrence of prostate cancer.

**Figure 8:**
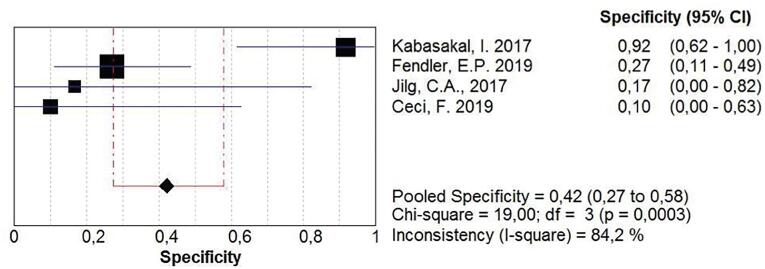
68Ga-PSMA-PET specificity for restating patients with biochemical recurrence of prostate cancer.

**Figure 9:**
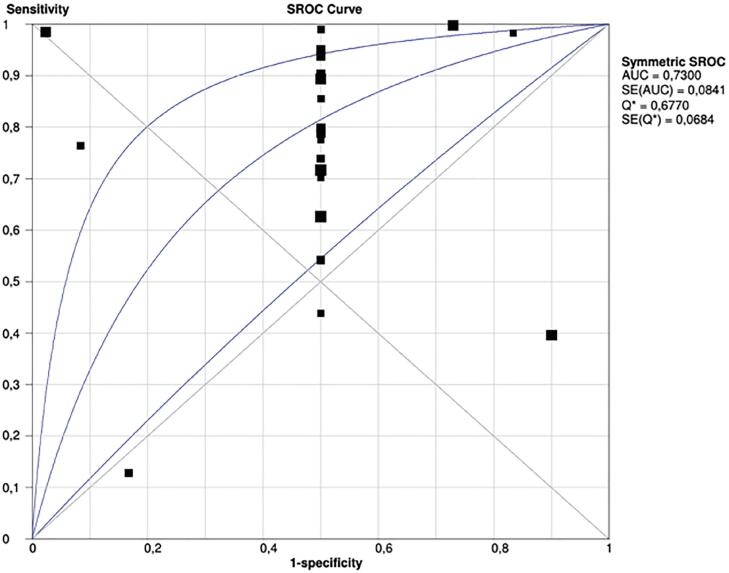
SROC curve for the pooled sensitivities and specificities on restating scenario, showing an area under the curve of 0.73.

The pool of studies that analyzed the power of ^68^Ga-PSMA PET in restaging recurrent prostate cancer resulted in a sensitivity of 0.76 (0.74 to 0.78), with heterogeneity of 96.7% (Figure-7). The specificity of the method, calculated based on the data available from the pool of studies, was 0.42 (0.27-0.58), and, again, a great inconsistency was noted (Figure-8). An sROC curve was plotted for those results, and the calculated area under the curve resulted in 0.73 (Figure-9).

When assessing biochemical recurrence, we observed that the higher the PSA level, the higher was the ability of the study to accurately demonstrate sites of accumulation of PSMA, positive for prostate cancer involvement. For patients with PSA LEVEL <0.5ng/mL, the positive LR pool estimation was 1.17 (0.37-3.73) and the sensitivity was 56% (0.42-0.68), increasing to 1.04 (0.38-2.85) and 58% (0.47-0.68), respectively, in patients with PSA between 0.5 and 1ng/mL. The positive probable pool ratio is 1.44 (0.59-3.51) and the sensibility is 81% (0.74-0.87) for patients with PSA 1 to<2ng/mL and, for patients with a PSA level higher than 2ng/mL, LR ratio is 1.78 (0.66-4.77), with sensitivity of 96% (0.93-0.97).

## DISCUSSION

Early biochemical recurrence represents the most clinically relevant subgroup of patients with relapsed prostate cancer, offering the possibility of potential curative salvage therapy concepts that might have a major impact on the outcome of patients. In biochemical recurrence, traditional imaging approaches (MRI, CT, and choline-based PET-CT) often fail to localize disease, mainly when the PSA level ranges lower than 2ng/mL. In our meta-analysis, we defined 4 subgroups based on PSA level: very low (<0.5ng/mL), low (0.5-1.0ng/mL), intermediate (1-2.0ng/mL), and high (>2.0ng/mL). We confirmed the value of ^68^Ga-PSMA-PET in all subgroups. Detection rates are indeed higher when PSA levels are higher; however, the ^68^Ga-PSMA-PET may have the greatest clinical impact in very low and low PSA level subgroups. To date, choline-based PET/CT is not recommended for patients with recurrent cancer and PSA level below 2ng/mL. Compared to choline-based PET/CT, evidence suggests that the ^68^Ga-PSMA PET has better sensitivity in detecting prostate cancer recurrence. On pooled analysis, the ^68^Ga-PSMA PET sensitivity was 56% for PSA under 0.5ng/mL. Our results for diagnostic accuracy are according to a recently published meta-analysis, reporting similar pooled sensitivity and specificity (3). To the date, most of the data outlining the utility of the ^68^Ga-PSMA PET is in the restaging for biochemical recurrence after definitive therapy.

Thus far, the value of ^68^Ga-PSMA PET in the accurate detection and delineation of intraprostatic tumor burden, which is important for diagnosis and treatment planning for patients with primary prostate cancer, is poorly explored. Although prostate cancer is mostly a multifocal disease, there is growing evidence that dominant intraprostatic lesions (DILs) may be responsible for metastatic and recurrent prostate cancer. Most of the ongoing studies use multiparametric magnetic resonance imaging (mpMRI); however, Zamboglou et al. compared seven patients who underwent mpMRI and ^68^Ga-PSMA PET before radical prostatectomy with co-registration between ^68^Ga-PSMA PET, mpMRI and histopathology. The sensitivity and specificity for ^68^Ga-PSMA PET were 75% and 87% and for mpMRI were 70% and 82%, respectively.

The present study highlights the possibility of improvement in evaluating prostate cancer, supporting the use of ^68^Ga-PSMA PET for diagnosing and staging. Traditionally, the primary staging of lymph nodes is performed using CT or MRI, but this relies on pathologic changes in lymph node morphology and size criteria. However, up to 80% of metastasis-involved nodes are smaller than the threshold limit of 8mm, typically used in clinical practice. Meta-analytical data for the traditional CT and MRI imaging approaches suggest sensitivity and specificity be 39-42% and 82%, respectively. A recent meta-analysis of choline-based tracers for PET-CT reviewed sensitivity of 49.2% and specificity of 95% for the detection of lymph nodes. Maurer et al. (34) performed a retrospective review of 130 patients undergoing high-risk prostate cancer, with a sensitivity of 65.9% and specificity of 98.9%. In our meta-analysis, the pool of studies showed the sensitivity and specificity were 93% and 87%, respectively, in detecting positive lymph nodes. As these are superior to those for traditional imaging, ^68^Ga-PSMA PET could allow complete and accurate diagnosing in primary staging compared to the current practice and potential improvement in patient care.

Despite significant advances, there is considerable scope for further research based on the use of ^68^Ga-PSMA PET. There is a need for more robust sensitivity and specificity data. In this meta-analysis, much of the histopathological correlation data available were not suitable for inclusion in our analysis for biopsy was performed according to clinician discretion. Selective lesion biopsy did not provide meaningful false-negative rates or specificity. Several groups reported the use of the ^68^Ga-PSMA PET for localization of intraprostatic malignancies, particularly in the context of focal therapies (50).

There are several limitations to this study. Firstly, most of the articles used for meta-analysis were derived from not measurable, retrospective, and single-institutional studies. The absence of more comprehensive studies could be justified as this technique is new and is still being explored for its inclusion in recent prostate cancer guidelines. Secondly, the heterogeneous nature of patient cohorts, treatment protocols, and studies used to pool sensitivity and specificity data, in particular, because most of the studies were carried with small sample size and patients were assessed in primary staging settings. Researchers should be encouraged to agree on data acquisition protocols and data analysis algorithms to increase the comparability of studies, which is one of the major issues in achieving evidence. Careful reporting of those methods may help evaluation to the extent that the results found to be applicable in other clinical settings.

^68^Ga-PSMA PET seems to provide higher sensitivity and specificity compared to alternative techniques. Our results reinforce the current evidence of the usefulness of ^68^Ga-PSMA PET, whereby the diagnostic evidence is more substantial in restaging with biochemical recurrence. The main applicability of ^68^Ga-PSMA PET certainly relies on restaging patients with biochemical recurrence after local treatment with curative intent.
